# Let-7d increases ovarian cancer cell sensitivity to a genistein analog by targeting c-Myc

**DOI:** 10.18632/oncotarget.20413

**Published:** 2017-08-23

**Authors:** Ying-Xia Ning, Xin Luo, Meng Xu, Xin Feng, Jian Wang

**Affiliations:** ^1^ Department of Gynaecology and Obstetrics, The First Affiliated Hospital of Guangzhou Medical University, Guangzhou 510120, China; ^2^ The First Affiliated Hospital of Jinan University, Guangzhou 510632, China; ^3^ Cancer Center, Traditional Chinese Medicine-Integrated Hospital, Southern Medical University, Guangzhou 510315, China; ^4^ Institute of Reproductive and Stem Cell Engineering, Central South University, National Engineering and Research Center of Human Stem Cell, Changsha, 41007, China

**Keywords:** ovarian cancer, c-Myc, let-7d, genistein analogue, PI3K/AKT

## Abstract

c-Myc is a key oncogenic transcription factor that participates in tumor pathogenesis. In this study, we found that levels of c-Myc mRNA and protein were higher in early ovarian cancer tissues than normal ovary samples. Increased c-Myc levels correlated positively with clinical stage I (Ia+b/Ic) in ovarian cancer patients. Patients with higher nuclear c-Myc expression had shorter overall survival times than patients with low c-Myc expression. Knocking down c-Myc sensitized ovarian cancer cells to 7-difluoromethoxyl-5,4’-di-n-octylgenistein (DFOG), a novel synthetic genistein analogue that suppressed PI3K/AKT signaling *in vitro* and *in vivo*. Finally, c-Myc was confirmed to be a direct target of let-7d, and let-7d-induced suppression of c-Myc increased the DFOG-sensitivity of ovarian cancer cells. These results indicate that nuclear c-Myc expression is an unfavorable factor in early ovarian cancer, and that let-7d increases ovarian cancer cell sensitivity to DFOG by suppressing c-Myc and PI3K/AKT signaling.

## INTRODUCTION

Ovarian cancer accounts for only about 3% of female malignancies, but causes more deaths than any other reproductive system cancer. The pathogenesis of ovarian cancer is complex and driven by dysregulation and aberrant activity of a variety of genes and signaling pathways [1, 2]. c-Myc encodes an oncogenic transcription factor that promotes cellular proliferation, migration, invasion, differentiation, and tumor stemness. It is a significant contributor to tumor pathogenesis, and elevated c-Myc expression is an unfavorable factor in many tumor types, including gastric [3], colorectal [4], prostate [5], breast [6], and ovarian [7].

Genistein (GEN) is a natural isoflavone in fruits, nuts, soybeans, and soy-based products. It induces cell apoptosis and cell cycle arrest in various cancer cells [8-10]. DFOG (7-difluoromethoxyl-5,4’-di-n-octylgenistein) is a novel synthetic genistein analogue that exhibits potent anticancer activity. In previous studies, we observed that DFOG markedly suppressed tumor stemness and promoted ovarian cancer cell apoptosis by downregulating FOXM1 and inducing FOXO3a [11-13].

Let-7 family miRNAs, including let-7d, reportedly suppress tumor pathogenesis [14-16]. On the other hand, Let-7d has been observed to contribute to the pathogenesis of some tumors. For example, Di et al observed that let-7d exerts both anti-oncogenic and oncogenic effects in osteosarcoma-derived 3AB-OS cancer stem cells [17]. In addition, let-7d reportedly directly targets HMGA2 [18], COL3A1, CCL7 and PBX3 [19-23], and thus suppresses tumor cell growth, migration, invasion, and metastasis. In this report, we describe our finding that elevated c-Myc is an unfavorable factor which promotes ovarian cancer pathogenesis, and that suppression of c-Myc by let-7d markedly sensitizes cancer cells to DFOG by downregulating the PI3K/AKT pathway.

## RESULTS

### c-Myc mRNA is upregulated in ovarian cancer

**Figure 1:**
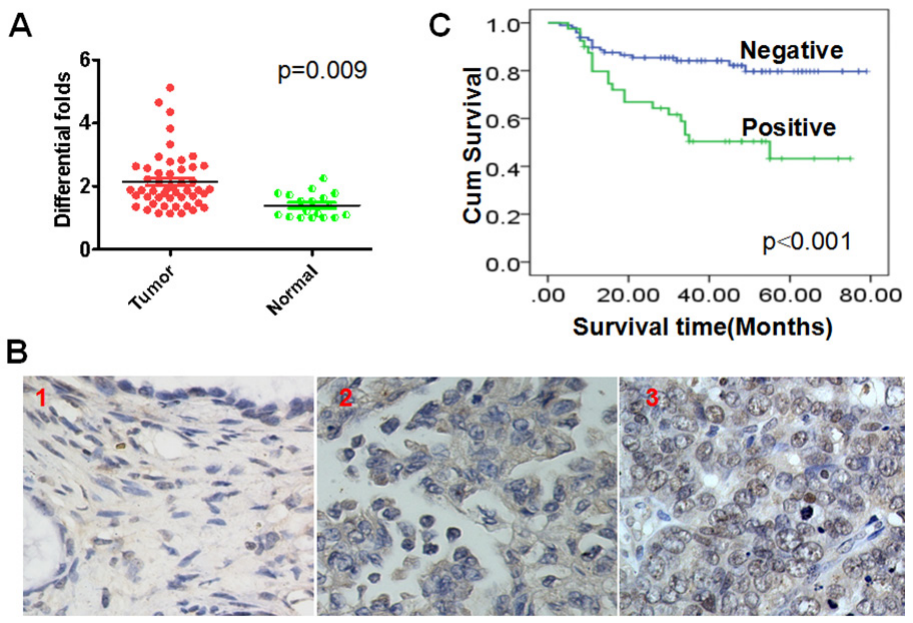
Elevated nuclear c-Myc expression is an unfavorable factor for ovarian cancer patients **(A)** c-Myc mRNA levels are downregulated in ovarian cancers as compared to normal ovarian tissues. **(B)** c-Myc protein expression in ovarian cancer and ovarian tissues. 1. Negative expression of c-Myc in normal ovarian; 2. Positive expression of c-Myc in the cytoplasm of ovarian cancer cells. 3. Positive expression of c-Myc in the nucleus of ovarian cancer cells. **(C)** Elevated nuclear c-Myc expression is an unfavorable factor for ovarian cancer patients.

When we examined c-Myc mRNA levels using real-time PCR, we observed that c-Myc transcription was significantly higher (p=0.009) in ovarian cancer tissues than normal ovarian tissues (Figure 1A). Moreover, nuclear staining in clinical samples showed that c-Myc expression at the protein level was also higher (p=0.031) in ovarian cancer than normal ovarian tissues (Figure 1B) (Table 1). These results suggest c-Myc likely plays a key role in the pathogenesis of ovarian cancer.

**Table 1:** Increased nuclear expression of c-Myc protein in ovarian cancer

		Nuclear c-Myc expression		
	N	Positive	Negative	*P* value
Ovarian cancer	138	40	98	
Ovarian tissues	47	6	41	0.031

### Correlation between clinicopathological features and nuclear c-Myc expression in stage I ovarian cancer

The correlation between nuclear c-Myc expression and clinical characteristics was also analyzed. As shown in Table 2, nuclear c-Myc expression did not correlate significantly with patient’s age, pathology classification, or grade degree in 138 stage I ovarian cancer cases (p>0.05). However, c-Myc expression correlated positively with clinical stage (Ia+b vs. Ic) (p=0.015).

**Table 2:** The correlation of Nuclear expression of c-Myc with clinical features in ovarian cancer

Clinical parameter		The expression of c-Myc		
	*n*	Positive expression (*n*)	Negative expression (*n*)	*P* value
age (year)				
≧50	58	17	41	1.0
<50	80	23	57	
Pathology classification				
S	107	34	73	0.261
M	31	6	25	
Grade				
Low	44	8	36	
Middle	64	20	44	0.111
High	30	12	18	
Clinical stage				
Ia+b	112	27	85	0.015
Ic	26	13	13	

*Kruskal-Wallis test

### Nuclear expression of c-Myc correlates negatively with overall survival time in stage I ovarian cancer patients

Kaplan-Meier analysis with the log-rank test was used to evaluate the prognostic value of c-Myc expression for stage I ovarian cancer patients. We observed that c-Myc expression correlated negatively with overall survival time. Patients who were positive for nuclear c-Myc expression had worse prognoses than those who were negative for c-Myc expression (Figure 1C) (p<0.001).

### Stable c-Myc knockdown suppresses pPI3K/AKT

**Figure 2:**
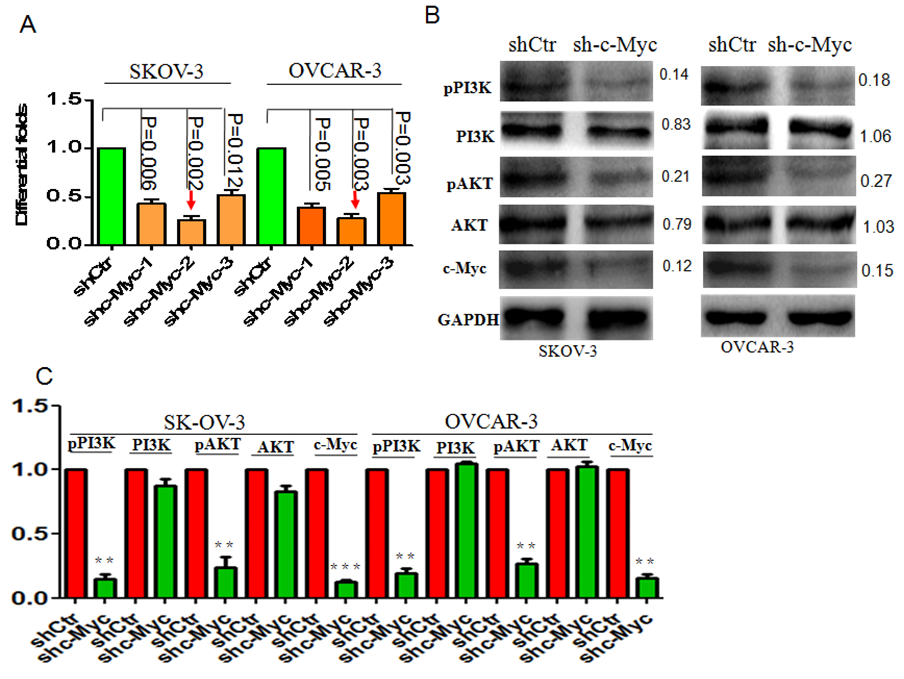
c-Myc knockdown suppresses pPI3K/AKT signaling in ovarian cancer **(A)** shRNAs were used to suppress c-Myc mRNA levels in ovarian cancer cells. **(B)** Suppression of c-Myc blocked pPI3K/AKT signaling, but not total PI3K/AKT protein levels. **(C)** Bar graph indicating the efficient suppression of pPI3K/AKT signaling after c-Myc knockdown. *p<0.05; **p<0.01; ***p<0.001;

**Figure 3:**
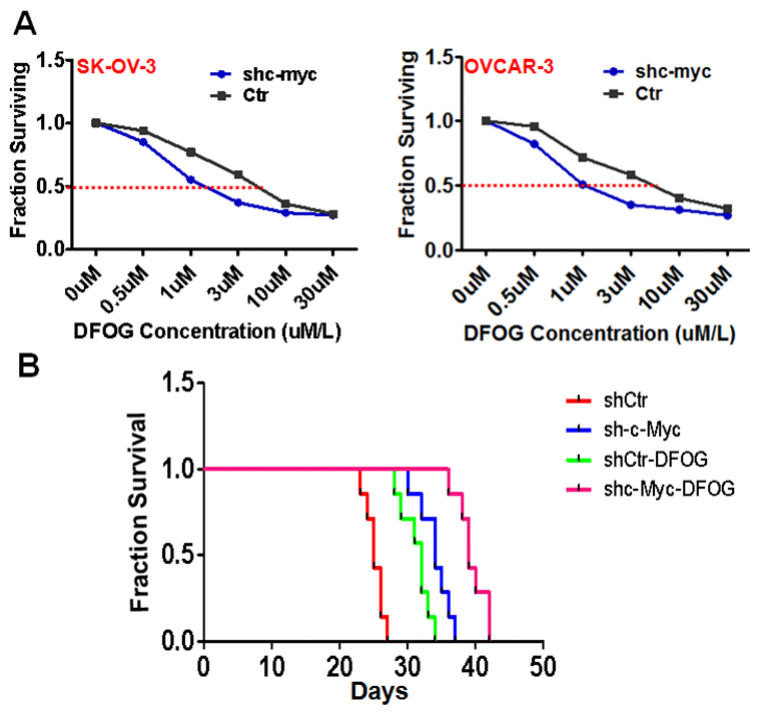
Suppression of c-Myc sensitizes ovarian cancer cells to DFOG *in vitro* and *in vivo* **(A** and **B)** Knocking down c-Myc induced ovarian cancer cell sensitivity to DFOG *in vitro*. **(C)** Suppression of c-Myc significantly sensitized ovarian cancer cells to DFOG *in vivo*.

Stable c-Myc knockdown in ovarian cancer cells was carried out using a lentiviral vector harboring shRNA-c-Myc. Real-time PCR analysis confirmed that c-Myc mRNA expression was suppressed in shRNA-c-Myc-2-expressing SKOV3 and OVCAR cells (Figure 2A). A corresponding decrease in c-Myc protein levels was confirmed by western blotting (Figure 3B). Interestingly, we observed that pPI3K/AKT signaling was significantly suppressed following c-Myc knockdown (Figure 2B, 2C).

### c-Myc suppression enhances ovarian cancer cell sensitivity to DFOG *in vitro*

DFOG is a novel synthetic genistein analogue that exerts anti-ovarian cancer effects by suppressing tumor stemness [8-10]. In the present study, we observed that knocking down c-Myc greatly sensitized ovarian cancer cells to DFOG. The IC50 of shMyc-expressing SKOV3 and OVCAR3 cells was significantly lower than that of their respective control parental lines (SKOV3: 1.12 ± 0.06μm vs 6.11 ± 0.20μm, p=0.003; OVCAR3: 1.56 ± 0.09μm vs 5.75 ± 0.29μm, p<0.001) (Figure 3A). This sensitizing effect was then examined *in vivo* with tumors originating from inoculated SKOV3 cells. Survival times for SKOV3 cell tumor-bearing nude mice were prolonged by DFOG treatment. Also exhibiting longer survival times were mice inoculated with c-Myc knockdown cells. And mice administered both c-Myc knockdown cells and DFOG treatment had the longest survival times (Figure 3B) (*P*<0.001). The average survival times for mice in control, shMyc, control+DFOG and shMyc+DFOG groups were 25.1, 34, 32.1 and 45.6 days, respectively.

### DFOG suppresses pPI3K/AKT and c-Myc signaling

**Figure 4:**
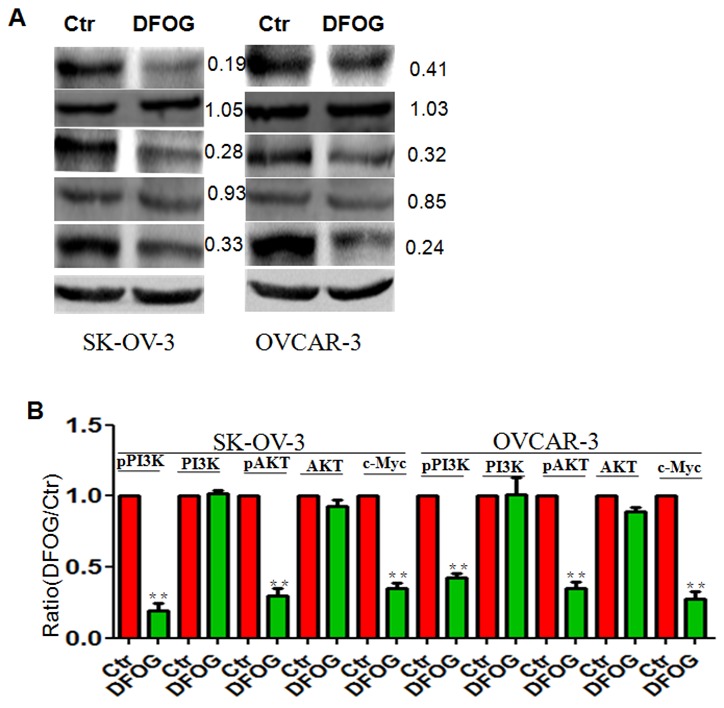
DFOG inhibits pPI3K/AKT and c-Myc signaling in ovarian cancer **(A)** pPI3K/AKT and c-Myc signaling were suppressed by DFOG. **(B)** Bar graph showing the efficient suppression of pPI3K/AKT and c-Myc signaling by DFOG. *p<0.05; **p<0.01;

Using DFOG to treat ovarian cells, we observed that levels of pPI3K/AKT pathway components were significantly reduced in parental SKOV3 and OVCAR cells. In addition, c-Myc was downregulated after DFOG treatment (Figure 4A, 4B).

### Let-7d induces DFOG sensitivity by directly targeting c-Myc

**Figure 5:**
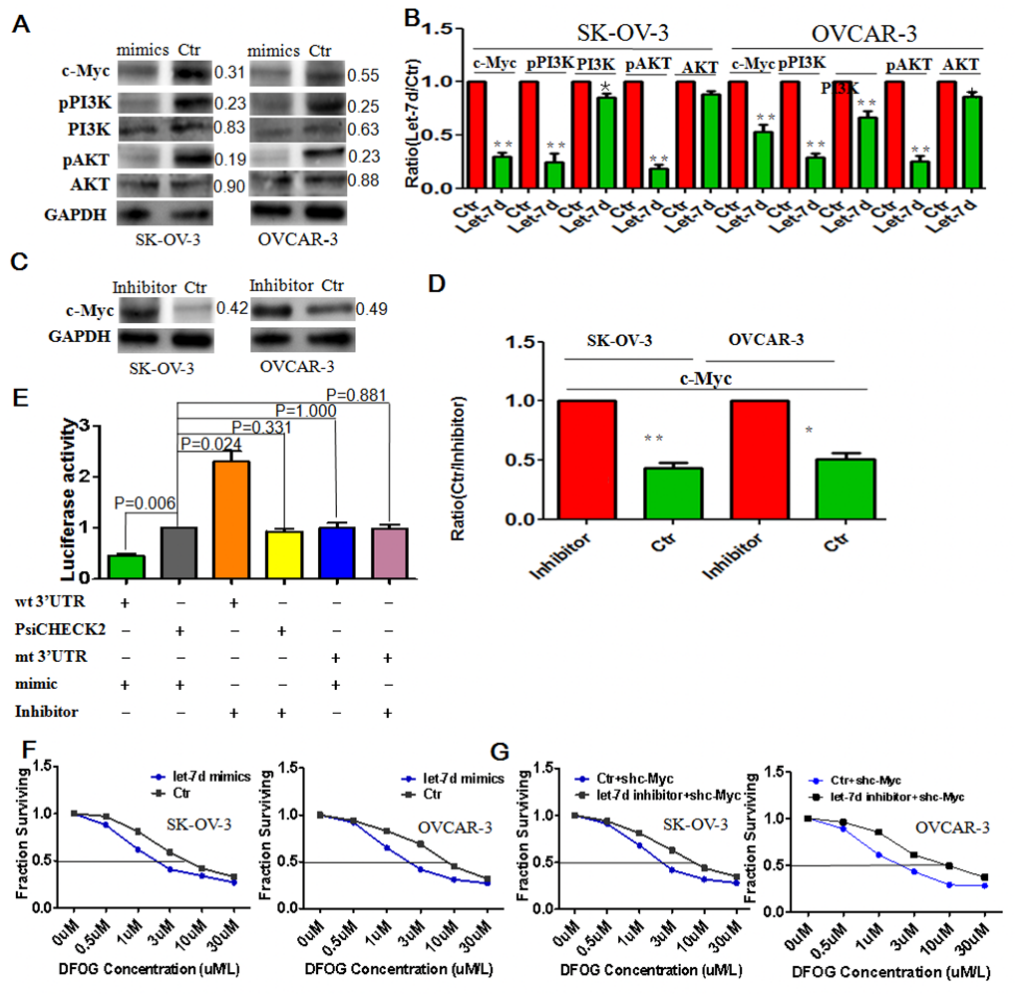
Let-7d directly targets c-Myc to reduce pPI3K/AKT signaling and increase the DFOG sensitivity of ovarian cancer cells **(A)** Let-7d suppressed c-Myc and in turn pPI3K/AKT signaling. **(B)** Bar graph showing the efficient suppression of pPI3K/AKT and c-Myc signaling by let-7d mimics. **(C)** Let-7d inhibitor restored expression of c-Myc protein. **(D)** Bar graph showing the increased efficiency of c-Myc expression induced by let-7d inhibitor. **(E)** Luciferase activity confirmed c-Myc to be a direct target of let-7d. **(F)** Let-7d induced DFOG sensitivity in ovarian cancer cells. G. Blocking let-7d with its inhibitor increased DFOG resistance in c-Myc knockdown ovarian cancer cells. *p<0.05; **p<0.01;

Let-7d mimics decreased c-Myc and pPI3K/AKT signals in ovarian cancer cells (Figure 5A, 5B), while Let-7d inhibitor exerted the opposite effects (Figure 5C, 5D). Luciferase activity indicated that c-Myc was a direct target of let-7d (Figure 5E). Moreover, let-7d mimics increased the sensitivity of ovarian cancer cells to DFOG, as evidenced by the lower IC50 levels (SKOV3: 1.12±0.19μm vs 5.80±0.35μm, p<0.001; OVCAR3: 2.31±0.29μm vs 8.52±0.57μm, p<0.001) (Figure 5F). Finally, we observed that blocking let-7d using its inhibitor increased the resistance to DFOG in shc-Myc-expressing ovarian cancer cells (SKOV3: 2.44±0.6μm vs 8.92±0.55μm, p<0.001; OVCAR3: 2.23±0.18μm vs 9.43±0.39μm, p<0.001) (Figure 5G).

### Let-7d is downregulated and negatively correlates with c-Myc expression

**Figure 6:**
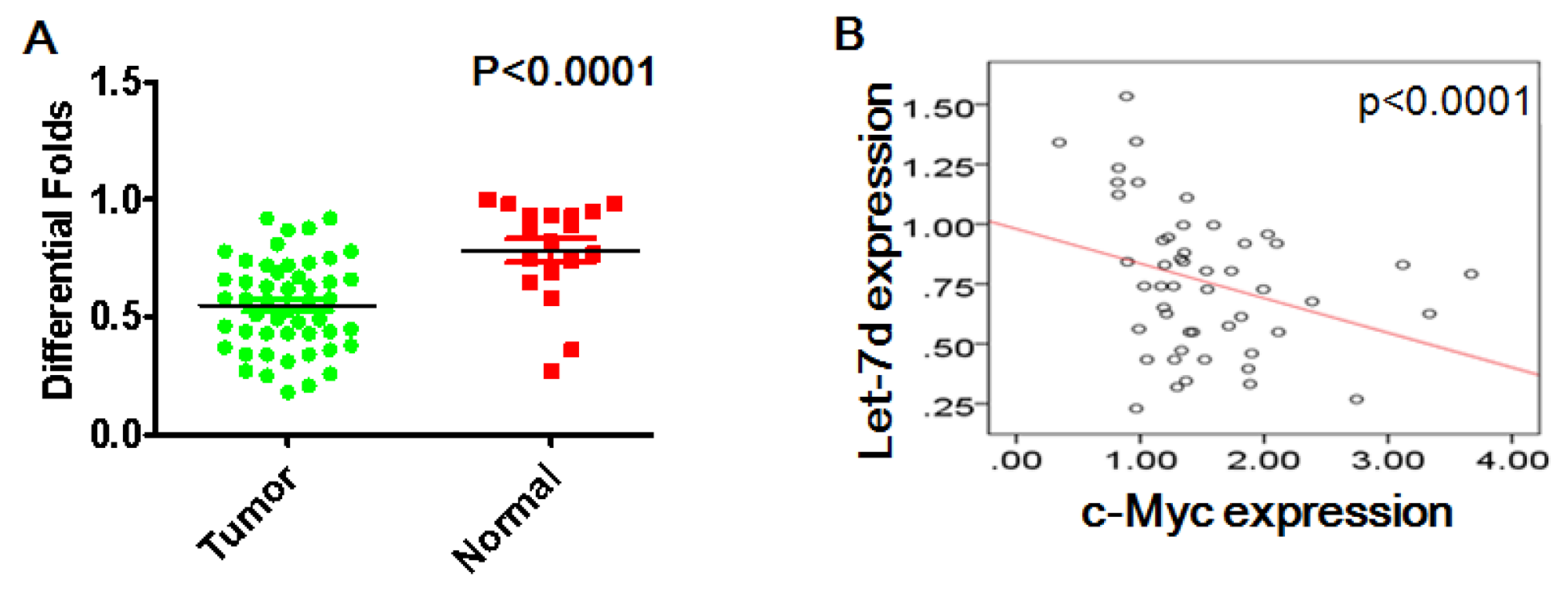
Let-7d levels correlate negatively with c-Myc in ovarian cancer **(A)** Let-7d expression was lower in ovarian cancer tissues than normal ovarian tissues. **(B)** Let-7d levels was correlated negatively with c-Myc expression in ovarian cancer

Real-time PCR assays indicated that let-7d expression was lower in ovarian cancer tissues than normal ovarian tissues (p <0.0001) (Figure 6A). Moreover, let-7d levels correlated negatively with levels of c-Myc mRNA in ovarian cancer tissues (Figure 6B)

## DISCUSSION

c-Myc is a conserved basic helix-loop-helix (HLH) leucine zipper transcription factor that induces cell growth, tumorigenesis, and tumor stemness. In the present study, we firstly observed higher c-Myc mRNA expression in ovarian cancer samples than normal ovarian tissues. This is consistent with an earlier report [7] and is indicative of the importance of c-Myc overexpression in the context of ovarian cancer.

c-Myc activity reportedly leads to dysregulation of numerous genes and thus promotes tumor initiation and progression [24, 25]. When we assessed the differential expression of c-Myc in stage I ovarian cancer and normal ovarian tissue, we found that nuclear expression of c-Myc was markedly higher in the cancer tissue. The finding is consistent with Zeng’s observation in prostate cancer [5], and further suggests that c-Myc participates in ovarian carcinogenesis. We also observed that although increased c-Myc did not correlate with age, pathology classification, or grade degree, it was positively related to clinical stage (Ia+b vs. Ic). This suggests upregulated c-Myc is also involved in early ovarian cancer progression.

Increased c-Myc expression has been documented as an unfavorable factor in prostate cancer [5]. We also observed that c-Myc correlates negatively with the overall survival time of stage I ovarian cancer patients. Patients exhibiting stronger c-Myc expression had shorter overall survival times than those of patients who lacked c-Myc expression. These data support the idea that c-Myc is a key oncogene in ovarian cancer.

Genistein, 5,7,4’-trihydroxylisoflavone, is a major component of soybean products and reportedly possesses anticancer activities. DFOG is a novel synthetic genistein derivative. We recently reported that DFOG suppresses cell growth and tumor stemness, and that it induces cell apoptosis by suppressing FOXM1 and upregulating FOXO3a in ovarian cancer [11-13]. c-Myc promotes chemotherapeutic resistance in tumors, including ovarian cancer [26-30]. Thus, suppression of c-Myc may be a way to elevate the sensitivity of tumor cells to drugs. Interestingly, we observed that knocking down c-Myc not only markedly elevated ovarian cell sensitivity to DFOG *in vitro*, but also greatly increased the survival time of tumor-bearing mice.

PI3K/AKT is a classical oncogenic signaling pathway, which promotes tumor pathogenesis and induces chemotherapy resistance [31-33]. Yu et al. observed that endoplasmic reticulum stress promotes autophagy and apoptosis and reverses chemoresistance of human small cell lung cancer cells by inhibiting the PI3K/AKT/mTOR signal. Zhao et al. found that miR-181c inhibits chemoresistance by suppressing PI3K/AKT in chronic myelocytic leukemia [34, 35]. In the present study, we observed that knocking down c-Myc expression led to reductions in PI3K/AKT signaling in ovarian cancer cells. This is in contrast to Kaur’s report that c-Myc suppresses PI3K/AKT signal in normal cells [36]. However, that observation has not been documented in the context of carcinogenesis and may reflect an inherent difference between normal and malignant cells. Similar to the effect of c-Myc knockdown, we found that DFOG treatment suppressed PI3K/AKT signaling, but also suppressed c-Myc expression. These results provide evidence that c-Myc suppression sensitizes ovarian cells to DFOG treatment.

Let-7d reportedly acts as a tumor suppressor participating in the pathogenesis of some tumors, including ovarian cancer. In previous documents, Let-7 (let-7b/c) contributed to HuR-mediated repression of c-Myc expression [37]. In the present study, we found that let-7d directly targets c-Myc and induced PI3K/AKT signaling to increase the DFOG sensitivity of ovarian cancer cells. We also observed that let-7d expression correlated negatively with c-Myc mRNA expression in ovarian cancers. These data demonstrate the importance of the let-7d/c-Myc signal in ovarian cancer.

In summary, our study has demonstrated that c-Myc mRNA levels are significantly elevated in ovarian cancer tissues. c-Myc expression correlates positively with clinical progression and is predictive of a poor prognosis in stage I ovarian cancer patients. Our data also demonstrate that c-Myc suppression sensitizes ovarian cancer cells to DFOG by inactivating PI3K/AKT signaling. Finally, c-Myc is a direct target of the tumor suppressor let-7d, and its suppression is associated with let-7d-mediated increases in DFOG-sensitivity in ovarian cancer.

## MATERIALS AND METHODS

### Cell culture and sample collection

The SKOV3 and OVCAR ovarian cancer cell lines were purchased from the Shanghai cell bank of the Chinese Academy of Sciences. The cells were maintained in RPMI-1640 medium supplemented with 10% fetal calf serum (FCS) (PAA Laboratories, Inc, Pasching, Austria). Tissues from patients included 50 fresh ovarian cancer samples and 18 normal ovarian samples. In addition, paraffin-embedded ovarian samples (138 clinical stage I cancers and 47 normal ovary) were obtained from the First Affiliated Hospital of Guangzhou Medical University. Clinical processes were approved by the hospital Ethics Committee, and patients gave informed written consent.

### RNA isolation and qRT-PCR

Total RNA was isolated using TRIzol reagent according to the manufacturer’s protocol (Invitrogen, USA), after which 1 μg of RNA was synthesized to cDNA using SuperScriptase III (Invitrogen) with random primers. PCR was performed using primers for c-Myc (sense: 5’GAGGAGAATGTCAAGAGGCG3’; antisense: ATAACTACCTTGGGGGCCTT) in accordance with the manufacturer’s instructions (Takara, Shiga, Japan). PCR reactions were repeated three times, and β-actin (sense: TGACGTGGACATCCGCAAAG; antisense: CTGGAAGGTGGACAGCGAGG) was used as an internal control.

### Immunohistochemistry

Immunohistochemistry was carried out as described previously [10] using a rabbit anti-human c-Myc polyclonal antibody (1:100 dilution; Santa Cruz Biotechnology, USA). Sections were stained using DAB, counterstained with hematoxylin, mounted in neutral gum, and visualized using a bright field microscope.

### Evaluation of staining

Stained tissue sections were reviewed separately by two pathologists blinded to the clinical parameters. Samples with ≥10% nuclear staining were considered positive for nuclear expression. Samples with <10% staining were considered negative for nuclear expression.

### Western blot analysis

Thirty-microgram aliquots of protein were subjected to 10% SDS-PAGE, after which the separated proteins were transferred to PVDF membranes. The membranes were then incubated first for 1-2 h in blocking solution, then for 1 h with the following primary antibodies: rabbit polyclonal c-Myc, PI3K, pPI3K, AKT, pAKT, and GAPDH (Cell Signaling Technology, Danvers, USA). An HRP-conjugated anti-rabbit IgG antibody was used as the secondary antibody (Zhongshan, Beijing, China). Signals were developed using enhanced chemiluminescence reagents (Pierce, Rockford, IL).

### Construction of stable c-Myc knockdown ovarian cancer cell lines

Four lentiviral vectors, three harboring shMycs and one harboring shCtrl, were prepared by Genechem Incorporation (Shanghai, China). These constructs were used to infect SKOV3 and OVCAR ovarian cancer cells, and polyclonal cells exhibiting GFP positivity were selected for further analysis using FACS.

### MTT cytotoxicity assay

shc-Myc SKOV3 and OVCAR ovarian cancer cells and their respective control cells were seeded into 96-well plates to a density of 5×10^3^ cells/well and then incubated in 100 μl DMEM supplemented with 10% FBS. Once the cells attached, 0, 0.5, 1, 3,10, or 30 μM DFOG was added to the wells, which were then incubated for 48 h at 37°C under 5% CO_2_. Thereafter, 10 μl of MTT (5 mg/ml) (Sigma, St. Louis, MO, USA) were added to each well, and plates were incubated for an additional 4 h at 37°C. The supernatants were then removed, and 100 μl of DMSO (Sigma) were added to each well. The optical density (OD) of each well at 490 nm was then measured. The calculated rates were then used for curve fitting and for calculating the half-maximal inhibitory concentration (IC50). Experiments were repeated three times.

### *In vivo* experiments in nude mice

*In vivo* experiments were approved by the Animal Care and Use Committee of Guangzhou Medical University. All mice were provided by the Central Animal Facility of Guangzhou Medical University at 4 weeks of age. Nude mice (BALB/C, nu/nu) were injected intraperitoneally with approximately 6×10^5^ c-Myc-silenced SKOV3 cells or control cells in 0.2 mL of buffered saline. Tumors were allowed to grow for 3 days, after which the animals were randomized into four groups (shCtr; shMyc; shCtr+DFOG; shMyc+DFOG), which were administered normal saline (NS) or DFOG (10 mg/kg) every 4 days. Survival was calculated using Kaplan Meier analysis.

### Transfection with let-7d mimics and its inhibitor

let-7d mimic and its inhibitor were purchased from Guangzhou RiboBio (RiboBio Inc, China). Ovarian cancer cells were seeded into a 6-well or 96-well plate (Nest, Biotech, China) to 40% confluence. After incubation for 24 h, the cells were transfected with let-7d mimics or its inhibitor using TurboFectTM siRNA Transfection.

### miRNA target validation

Based on analysis using miRwalk software (University of Heidelberg, Mannheim, Germany), c-Myc was predicted to be a direct target of let-7d. A 256-base fragment of the wild-type c-Myc 3’UTR with a let-7d binding site was amplified and then cloned into psiCHECK-2 vector (WT) containing XhoI and NotI restriction enzyme sites. Site-directed mutagenesis of the let-7d binding site in c-Myc 3’UTR was performed using a GeneTailor System (Invitrogen), and its cloning into psiCHECK-2 yielded the mutant-type vector MT. For reporter assays, WT, MT or control psiCHECK-2 vector were cotransfected into ovarian cancer cells with let-7d mimics or inhibitor. Luciferase activity was measured 48 h after transfection using a Dual-Luciferase Reporter Assay System (Promega Corporation, Madison, WI, USA).

### Statistical analysis

All data were analyzed for statistical significance using SPSS 13.0 software and presented as the mean ± SD. Values of P < 0.05 were considered statistically significant. Student’s t-test was applied to examine differences in c-Myc mRNA expression between normal and ovarian cancer tissues. One-way ANOVA was used to determine the differences in between groups in all *in vitro* analyses.

## Abbreviations

DFOG: 7-difluoromethoxyl-5,4’-di-n-octylgenistein
GEN: Genistein
qRT-qPCR: Quantification reverse transcription-quantitative polymerase chain reaction
